# Supplementary material for: An open-source framework for large-scale, flexible evaluation of biomedical text mining systems
Source: J Biomed Discov Collab. 2008 Jan 29;3:1. doi: 10.1186/1747-5333-3-1 (PMC2276192; doi:10.1186/1747-5333-3-1)
Supplement: Additional file 1 — Evaluation platform source code. The additional file contains the software evaluation framework discussed in this paper. The most current release can be downloaded from SourceForge.net [1]. See the accompanying README file for instructions on installation and use. Also included in the distribution is a collection of UIMA wrappers for some commonly used BioNLP tools and annotated corpora. [file 1747-5333-3-1-S1.zip › bionlp_uima_repository-1.0.1/docs/api/edu/uchsc/ccp/uima/util/opendmap/ReferenceProcessor.html]

ReferenceProcessor (Center for Computational Pharmacology Util/UIMA API)


|  |  |  |
| --- | --- | --- |
| **Overview** \| **Package** \| **Class** \| **Tree** \| **Deprecated** \| **Index** \| **Help** | |  |
| **PREV CLASS**   **NEXT CLASS** | **FRAMES**    **NO FRAMES**     **All Classes** |  |
| SUMMARY: NESTED \| FIELD \| CONSTR \| METHOD | DETAIL: FIELD \| CONSTR \| METHOD |  |


---


## edu.uchsc.ccp.uima.util.opendmap Class ReferenceProcessor

```
java.lang.Object
  edu.uchsc.ccp.uima.util.opendmap.ReferenceProcessor
```

---

``` public class ReferenceProcessor extends Object ```

Get the the desired references that DMAP has recognized. Order
them according to a simple score that prefers references that
include every word over the span that was used to recognize
them.

**Author:**
:   R. James Firby

---

**Constructor Summary**

`ReferenceProcessor()`


| **Method Summary** | |
| --- | --- |
| `static double` | `calculateScore(edu.uchsc.ccp.opendmap.Reference r)`             Score the reference. |
| `static Collection<edu.uchsc.ccp.opendmap.Reference>` | `getRecognizedReferences(edu.uchsc.ccp.opendmap.Parser parser, Collection<String> interests, boolean debug)`             Get the desired references ordered by score. |

**Methods inherited from class java.lang.Object**

`clone, equals, finalize, getClass, hashCode, notify, notifyAll, toString, wait, wait, wait`

**Constructor Detail**

### ReferenceProcessor

```
public ReferenceProcessor()
```


**Method Detail**

### getRecognizedReferences

```
public static Collection<edu.uchsc.ccp.opendmap.Reference> getRecognizedReferences(edu.uchsc.ccp.opendmap.Parser parser,
                                                                                   Collection<String> interests,
                                                                                   boolean debug)
```

:   Get the desired references ordered by score.

    :   **Parameters:**: `parser` - The parser that has processed an utterance and is holding the references.: `interests` - The references of interest. A collection of reference types.: `debug` - True if detailed debugging information should be printed. **Returns:**: The ordered list of references of interest.

---


### calculateScore

```
public static double calculateScore(edu.uchsc.ccp.opendmap.Reference r)
```

:   Score the reference.

    :   **Parameters:**: `r` - The reference **Returns:**: The score


---


|  |  |  |
| --- | --- | --- |
| **Overview** \| **Package** \| **Class** \| **Tree** \| **Deprecated** \| **Index** \| **Help** | |  |
| **PREV CLASS**   **NEXT CLASS** | **FRAMES**    **NO FRAMES**     **All Classes** |  |
| SUMMARY: NESTED \| FIELD \| CONSTR \| METHOD | DETAIL: FIELD \| CONSTR \| METHOD |  |


---
